# Automated Three-Dimensional Imaging and Pfirrmann Classification of Intervertebral Disc Using a Graphical Neural Network in Sagittal Magnetic Resonance Imaging of the Lumbar Spine

**DOI:** 10.1007/s10278-024-01251-2

**Published:** 2024-09-12

**Authors:** David Baur, Richard Bieck, Johann Berger, Patrick Schöfer, Tim Stelzner, Juliane Neumann, Thomas Neumuth, Christoph-E. Heyde, Anna Voelker

**Affiliations:** 1 Department for Orthopedics, Trauma and Plastic Surgery, University Hospital Leipzig, Liebigstraße 20, 04103 Leipzig, Germany; 2 Innovation Center Computer Assisted Surgery (ICCAS), Universität Leipzig, Leipzig, Germany

**Keywords:** Artificial intelligence, Convolutional neural network, Graph neural network, Machine learning, MRI, Spine imaging, Pfirrmann classification, Intervertebral disc degeneration

## Abstract

This study aimed to develop a graph neural network (GNN) for automated three-dimensional (3D) magnetic resonance imaging (MRI) visualization and Pfirrmann grading of intervertebral discs (IVDs), and benchmark it against manual classifications. Lumbar IVD MRI data from 300 patients were retrospectively analyzed. Two clinicians assessed the manual segmentation and grading for inter-rater reliability using Cohen's kappa. The IVDs were then processed and classified using an automated convolutional neural network (CNN)–GNN pipeline, and their performance was evaluated using F1 scores. Manual Pfirrmann grading exhibited moderate agreement (κ = 0.455–0.565) among the clinicians, with higher exact match frequencies at lower lumbar levels. Single-grade discrepancies were prevalent except at L5/S1. Automated segmentation of IVDs using a pretrained U-Net model achieved an F1 score of 0.85, with a precision and recall of 0.83 and 0.88, respectively. Following 3D reconstruction of the automatically segmented IVD into a 3D point-cloud representation of the target intervertebral disc, the GNN model demonstrated moderate performance in Pfirrmann classification. The highest precision (0.81) and F1 score (0.71) were observed at L2/3, whereas the overall metrics indicated moderate performance (precision: 0.46, recall: 0.47, and F1 score: 0.46), with variability across spinal levels. The integration of CNN and GNN offers a new perspective for automating IVD analysis in MRI. Although the current performance highlights the need for further refinement, the moderate accuracy of the model, combined with its 3D visualization capabilities, establishes a promising foundation for more advanced grading systems.

## Introduction

Low back pain (LBP) is a prevalent condition often associated with degenerative lumbar spinal diseases. These degenerative changes primarily affect the intervertebral disc (IVD) region, involving complex alterations in both the nucleus pulposus and adjacent vertebral end plates [1]. The Pfirrmann classification system, introduced in 2001, has been widely used to standardize the description of these degenerative changes [2]. This system categorizes IVD degeneration into five progressive severity levels based on the disc's structural integrity, signal intensity variations, and alterations in disc height.

However, the Pfirrmann classification system has shown limitations in terms of reliability and consistency. Studies have reported moderate intra-observer agreement with kappa values ranging from 0.69 to 0.81, while inter-rater reliability metrics have been found to vary between 0.491 and 0.830 [3, 4]. These inconsistencies pose challenges for the seamless integration of this classification into routine clinical diagnostics.

In response to these limitations, researchers have developed alternative classification systems. For instance, Soydan et al. proposed a novel 8-grade system that aims to overcome some of the limitations of the Pfirrmann classification by providing a more comprehensive assessment of disc degeneration, including aspects such as disc homogeneity, nucleus-annulus separation, signal intensity, disc height, and disc border regularity [5].

A recent meta-analysis by Compte et al. critically evaluated the performance of various machine learning algorithms in identifying lumbar disc degeneration and associated pathologies from MRI, comparing them to radiologists. The study highlighted that while current models, particularly deep learning approaches, show promise, they often underperform in external validations, pointing to the need for further research in developing robust algorithms that can generalize across diverse datasets [6].

Recent advancements in artificial intelligence (AI) and machine learning (ML) have opened new ways to address these limitations. Several studies have explored the application of ML techniques in automating the assessment of disc degeneration. For instance, Niemeyer et al. developed a deep learning model for the classification of disc degeneration based on MRI data, achieving promising results with an accuracy of 97.3% [7]. Their convolutional neural network (CNN) approach demonstrated high reliability across different spinal levels and degenerative stages.

Graph Neural Networks (GNNs) have emerged as a promising tool for processing non-standardized and irregular data, making them particularly suitable for 3D medical image analysis. Zhang et al. reviewed the application of GNNs in image-guided disease diagnosis, highlighting their ability to effectively represent relationships between relevant regions of interest in medical images [8]. Further supporting this, Ahmedt-Aristizabal et al. systematically reviewed the use of GNNs in medical diagnosis and analysis, emphasizing the potential of GNNs to capture both local and global structural information, which is critical in medical image analysis [9].

Despite these advancements, several gaps and limitations persist in the current literature. Most studies focus on 2D image analysis, potentially overlooking important 3D structural information. The integration of CNN and GNN approaches for comprehensive IVD analysis has not been thoroughly investigated, and the potential of 3D point-cloud representations in enhancing the accuracy of IVD degeneration classification remains unexplored.

To address these gaps, our study aims to develop and evaluate a novel CNN-GNN pipeline for automated 3D imaging and Pfirrmann classification of IVDs. By leveraging both 2D and 3D information, we hypothesize that this approach could provide more accurate and consistent assessments of IVD degeneration, potentially overcoming the limitations of current classification methods.

## Materials and Methods

This study hypothesized that incorporating three-dimensional (3D) geometric forms to differentiate degenerative IVD pathologies could more accurately distinguish between degeneration classes of IVDs. Transitioning from conventional convolutional neural networks (CNNs), such as Pfirrmann class prediction, to GNNs is a potentially promising solution. We decided to train a GNN and compare its grading capabilities for Pfirrmann classes with those of human researchers.

### Study Subjects

As this study was designed retrospectively, approved by the local ethics committee (025/21-ek), and was performed in accordance with the Declaration of Helsinki, the need for informed consent was waived. Inclusion was limited to patients with LBR who underwent MRI diagnostics with sagittal T2-weighted magnetic resonance imaging (MRI) slices of the whole lumbar spine (L1-S1) at our university hospital in 2015–2021. The exclusion criteria were age < 18 years, previous lumbar spine surgery, pre-existing spine implants, detectable tumors, or fractures. In total, 300 patients with 6043 sagittal T2-weighted turbo spin-echo (TSE) MR scans of the lumbar spine were included.

### MRI Imaging

Different MRI scanners were used in the timeframe of patient inclusion ((1.5 Tesla MRI Aera, 3.0 Tesla Trio, Siemens, Erlangen, Germany) or (3.0 Tesla MRI Ingenia, Philipps, Amsterdam, Netherlands)). PACS software Syngo Plaza (Siemens Medical Solutions, Germany) was used. All sagittal T2-weighted TSE sequences of the lower spine were included. There were no exclusions based on the imaging quality.

### Generation of Ground Truth Labels and csv Files

We extracted sagittal T2-weighted MRI slices of the lumbar spine from all 300 patients. After pseudonymization of the patient data, MRI slices were saved in the DICOM format. For mask generation, two independent spine surgery specialists segmented the data using segmentation software (Materialize – Mimics Version 22.0.0.524 Löwen, Belgium). The MRI slices were segmented into labels for each lumbar vertebra (L1-S1) and lumbar IVDs (L1/2-L5/S1) within all sagittal slices. All midline sagittal MR images of the lumbar spine were graded according to the Pfirrmann classification. The results were saved in Microsoft Excel. Intra- and inter-rater reliabilities were calculated using Cohen's kappa coefficients [10]. All labels were saved as 3-channel RGB. jpg files with a resolution of 300 dots per inch. For training, a.csv file was created. It contained labels for vertebrae, IVDs, and Pfirrmann grading of all respective IVDs for direct implementation in a Python 3.6 environment to prepare the data for further processing.

### Image Processing Pipeline and Deep Learning Task

We automated an image-processing pipeline that accepted patient-individual sagittal MRI slices as input and classified IVD information according to a previously applied Pfirrmann grading. The pipeline consists of three main stages: (1) CNN-based image segmentation using a U-Net architecture to segment IVDs and vertebral bodies from each MRI slice, (2) 3D surface and volume reconstruction, where segmented slices were combined to create 3D representations of each IVD using marching cubes algorithm and point cloud generation techniques, and (3) graph-based classification, where the 3D point cloud representations were converted into graphs and analyzed using a GNN to predict Pfirrmann grades.

Our dataset consisted of multiple MRI slices per patient. To account for the potential correlation between slices from the same patient, we implemented a hierarchical approach in our analysis. For the segmentation task, we used a patient-level cross-validation strategy, ensuring all slices from a single patient were either in the training set or the test set, but never split between the two. For the classification task, we aggregated predictions across all slices for each patient before making a final classification decision.

### MRI Segmentation

**Fig. 1:**
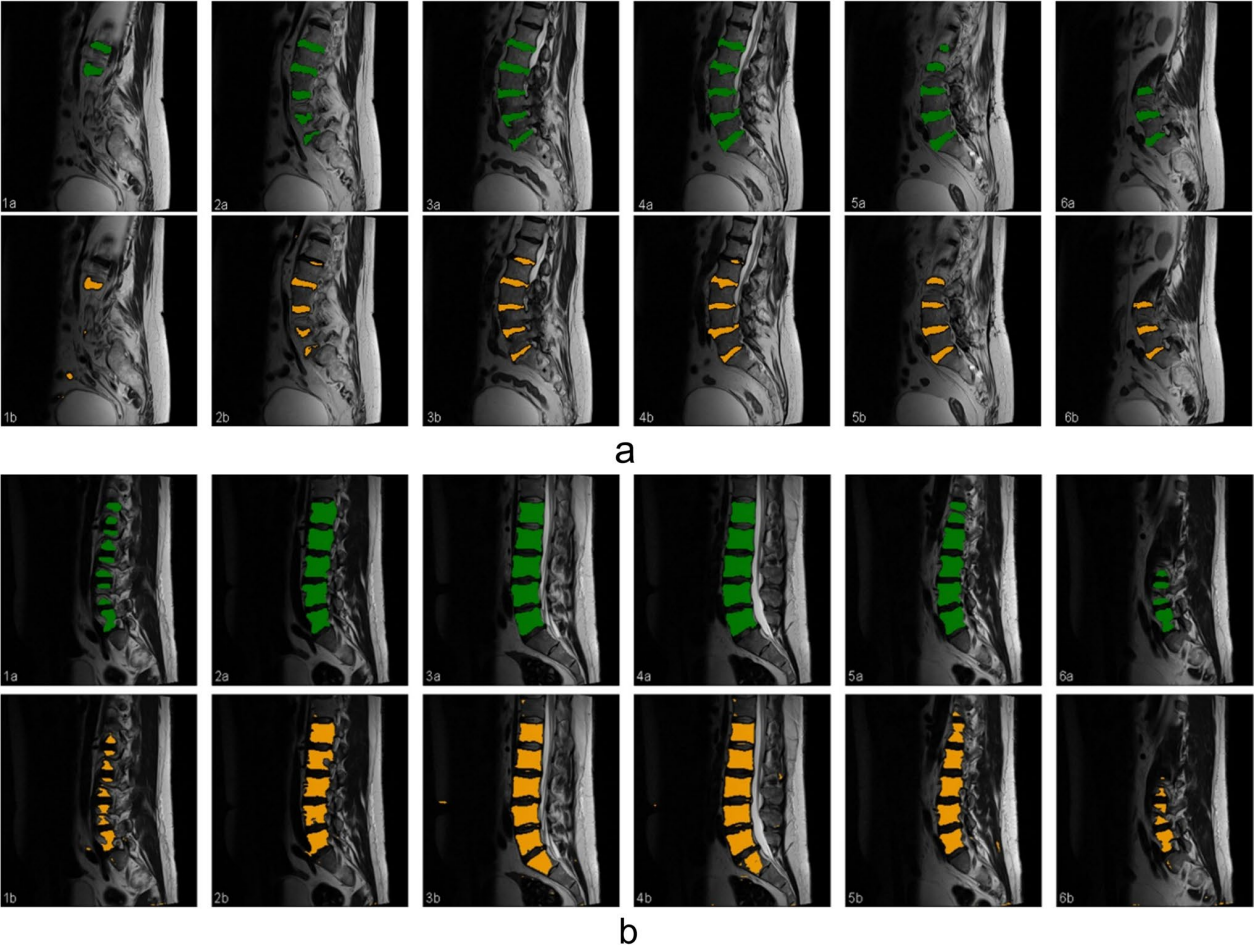
**a** The figure shows example MRI images of the lumbar spine from a single patient, illustrating ground truth (top row, a) and U-Net segmentation masks (bottom row, b) of intervertebral discs. The segmentation metrics (IoU, F1, Precision, Recall) for each pair of images are as follows: 1 (0.74, 0.82, 0.87, 0.78), 2 (0.82, 0.89, 0.94, 0.85), 3 (0.91, 0.95, 0.95, 0.95), 4 (0.86, 0.92, 0.94, 0.90), 5 (0.91, 0.95, 0.96, 0.95), and 6 (0.96, 0.98, 0.97, 0.98). **b** The figure shows MRI images of the lumbar spine from a single patient, illustrating ground truth (top row, a) and U-Net segmentation masks (bottom row, b) of vertebra bodies. The segmentation metrics (IoU, F1, Precision, Recall) for each pair of images are as follows: 1 (0.78, 0.86, 0.87, 0.85), 2 (0.92, 0.96, 0.96, 0.95), 3 (0.91, 0.95, 0.92, 0.98), 4 (0.94, 0.97, 0.95, 0.99), 5 (0.93, 0.96, 0.97, 0.96), and 6 (0.89, 0.94, 0.91, 0.97)

For segmentation, a pre-trained U-Net model (feature size = 32), previously employed for the identification of muscle and fat tissue in axial images, was used [11, 12]. The model was fine-tuned on the segmentation labels generated for the axial MRI slices as in the previous studies. We initiated a ten-fold cross-validation study as part of the training process. The slices and labels were transformed and augmented to increase the training sample size and reduce overfitting. The segmentation performance was evaluated using the F1 score (Fig. 1a, b).

For the segmentation model, we employed a tenfold cross-validation approach. In each fold, 10% of the data was used as test data, while the remaining 90% was split into 85% training and 15% validation data. We used an Adam optimizer with a learning rate of 0.001. The loss function used was dice loss, which is particularly effective for segmentation tasks.

Segmentation was performed on all available sagittal slices for each patient, not just the midline. This comprehensive approach allowed us to construct a detailed 3D representation of each IVD, capturing its full geometry across multiple slices.

### 3D Surface and Volume Reconstruction

**Fig. 2:**
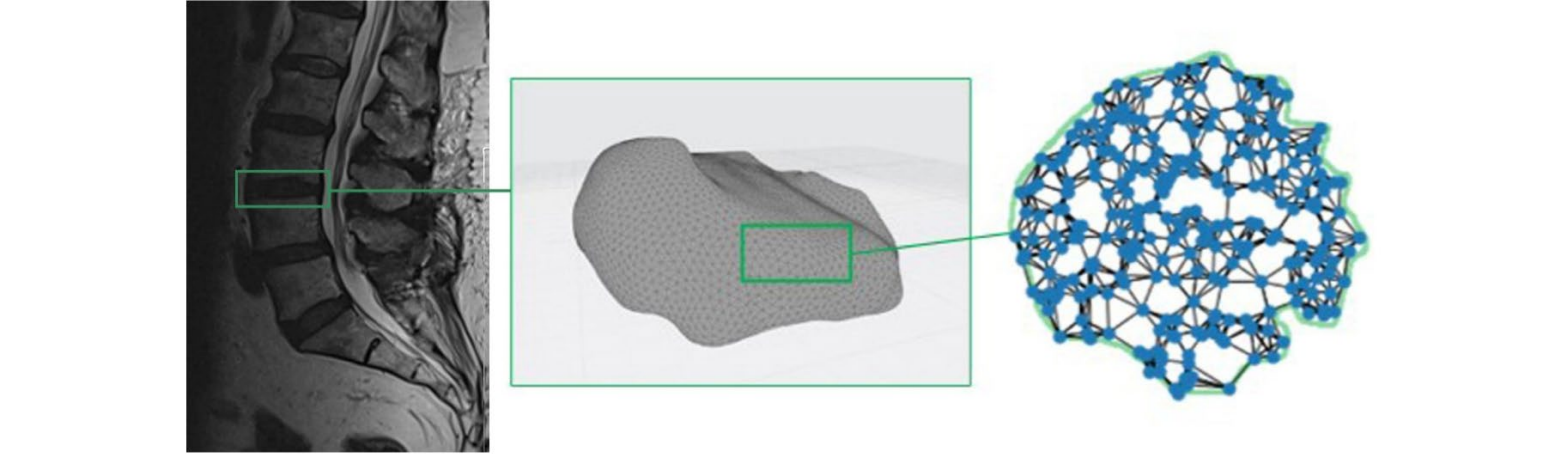
Overview of 3D reconstruction of the native IVD after automatic segmented MRI slices into 3D point cloud representation of a target intervertebral disc

In the reconstruction step, the segmented MRI slices were processed into 3D point-cloud representations of the surface and volume of the target IVDs for further analysis (Fig. 2). This first required the reconstruction of voxel representations of segmented MRI slices based on known pixel spacing from the DICOM imaging parameters [13]. An MRI volume can be represented as a 3D array, where each voxel's position is defined by its row, column, and slice indices (x, y, z coordinates), and its intensity value represents the measured signal at that location. This representation allows us to treat the MRI volume as a discrete scalar field for subsequent processing steps.

To capture the 3D geometry of the IVDs more flexibly, we employed random sampling of 80% of the points within the mesh volume, intentionally deviating from the regular grid structure of the original MRI voxels. This method has been shown to improve the representation of complex shapes and boundaries by allowing for a more adaptive structure, which is beneficial for graph-based analysis in 3D medical imaging [14, 15]. The resulting surface meshes comprised vertices and edge connections and were further processed to remove artifacts and improve the overall mesh quality using feature-preserving smoothing and edge splitting. This resulted in minimal reduction in the ground-truth mesh volume. Subsequently, point sampling was performed using the volume of the consolidated surface meshes. We applied the following approaches: (a) native voxel-to-volume, (b) random, and (c) regularized ellipsoid sampling. Using (a), we translated the scalar field values from the DICOM slices into node intensities; (b) was based on rejection sampling from inside the mesh; and (c) was based on the maximum and minimum values of the disc magnitudes.

Normalization to [-1,1] was applied to the data, and feature calculations were performed. These calculations included the distance of the nodes to the disc center, analogous to the nucleus, and the distance of the nodes to specified hypernodes that represent the point of maximum distance in each direction [16]. Positional encoding with a Laplacian eigenvector for each node with a step size of 20 and positional encoding with a random walk step size of 20 were used.

A radius graph (0.1 radius) was used to generate edge indices between nodes to perform message passing, and face indices were employed as edge indices [17]. Two reference architectures were used for comparison. First, a standard GCN model performing convolutions over the node features was used as the baseline [18]. The second model used two subnetworks that employed GCN and x-convolution layers separately, combined with attention layers for consolidation [19]. This dual-graph model incorporated the advantages of both convolution operations over feature maps and the spatial operations of x-conv across point clouds.

### Model Training and Optimization

To train and optimize the dual-graph model, we implemented various augmentation functions, including random rotation and random jitter; this was aimed to increase the diversity of the training dataset and to improve the model generalization [20]. The model was trained for 50 epochs using decoupled weight decay regularization with an AdamW optimizer [21].

To address the issue of class imbalance, we employed a weighted geometric mean of the focal loss and cross-entropy loss (CEL) as the objective function for model optimization [22]. The weights were adjusted to offset class imbalance and ensure adequate representation of all IVD classes during the training process.

### Statistical Analysis

We evaluated the inter-rater reliability of the IVD classification among human experts (two spine surgeons) and a dual-graph model using Cohen's kappa coefficient [10]. Further, we computed Fleiss' kappa to assess reliability among all three raters [23]. The model performance metrics, including precision, recall, and F1 score were calculated to assess the effectiveness of the dual-graph model in classifying IVDs according to the Pfirrmann grading.

## Results

A total of 300 patients were included in the study, yielding 6043 sagittal T2-weighted TSE MR scans of the lumbar spine. The descriptive statistics of the patients are presented in Table 1.

**Table 1:** Descriptive patients’ data

Characteristic	All patients (*n* = 300)	Women (*n* = 145)	Men (*n* = 155)
Age (mean, range)	58.94 (15–92)	59.9 (15–92)	58 (16–86)
Weight (kg) (STD)	78.5 (16.2)	72.3 (15.8)	84.2 (14.7)
Pfirrmann score distribution (*n*, %)			
Grade I	225 (15%)	108 (14.9%)	117 (15.1%)
Grade II	405 (27%)	194 (26.8%)	211 (27.2%)
Grade III	450 (30%)	220 (30.3%)	230 (29.7%)
Grade IV	300 (20%)	145 (20%)	155 (20%)

Automated image segmentation using the U-Net model demonstrated high efficiency. For the predicted segmentation of the IVD, F1 score, precision, and recall of 0.85, 0.83, and 0.88, respectively were achieved. Similarly, high values were obtained for the predicted segmentation of the vertebral bodies, with F1 score, precision, and recall of 0.87, 0.87, and 0.89, respectively. These results reflect the model's performance at the slice level, while accounting for patient-level correlations through our hierarchical approach as described in the methods.

Analysis of the Pfirrmann classification revealed moderate agreement between the two researchers across all lumbar disc levels. The mean Cohen's kappa value for inter-rater reliability was 0.507. The Cohen's kappa values for each IVD were as follows: for L1/2, L2/3, L3/4, L4/5, L5/S1, κ = 0.455, 0.500, 0.519, 0.496, and 0.565, respectively. These kappa values indicated that the inter-rater agreement exceeded what would be expected by chance alone and fell within the "moderate agreement" category.

The highest agreement was observed at the L5/S1 level, suggesting that the researchers were more consistently aligned in their assessments of the lower lumbar IVDs. Conversely, the L1/2 level exhibited the lowest agreement, indicating greater variability in grading this particular disc level.

Further analysis of the grading outcomes revealed that most gradings were consistent between the two researchers, with exact matches ranging from 131 to 207 across spinal levels, and single-grade differences being more common than two or more grade differences.

The performance of the GNN model in classifying the same disc degeneration was assessed using precision, recall, and F1 score metrics. Detailed analyses are presented in Tables 2, 3, and 4. The model achieved the highest precision (0.81), recall (0.63), and F1 score (0.71) at L2/3. The mean precision, recall, and F1 scores of the model across all levels were 0.46, 0.47, and 0.46, respectively. The standard deviations for these metrics across levels indicated variability in the model's performance, in the range 0.058–0.259, 0.052–0.213, and 0.081–0.209 for precision, recall, and the F1 score, respectively.

**Table 2:**
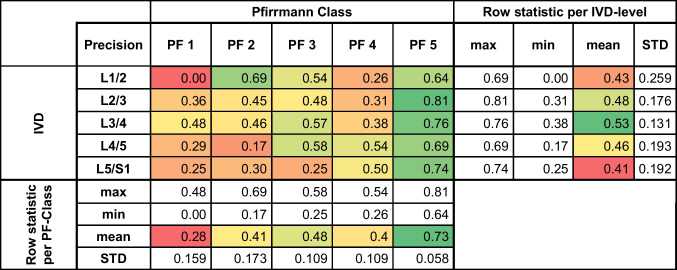
Precision graph model

The performance of the GNN model in classifying disc degeneration was assessed using precision metric. The metric values are presented as a heat map, indicating the correlation between each intervertebral disc and the predicted Pfirrmann classification

*IVD* intervertebral disc, *PF* Pfirmann

**Table 3:**
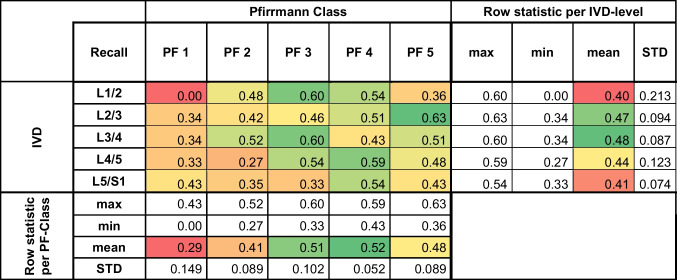
Recall graph model

The performance of the GNN model in classifying disc degeneration was assessed using recall metric. The metric values are presented as a heat map, indicating the correlation between each intervertebral disc and the predicted Pfirrmann classification

*IVD* intervertebral disc, *PF* Pfirmann

**Table 4:**
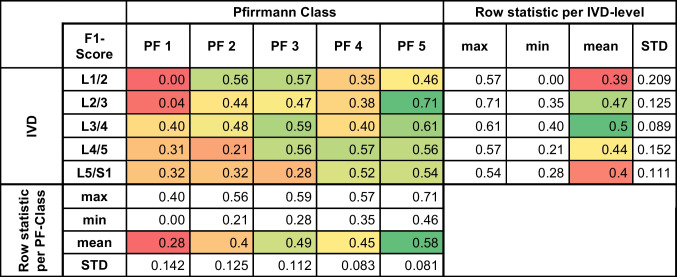
**F1** score graph model

The performance of the GNN model in classifying disc degeneration was assessed F1 score metric. The metric values are presented as a heat map, indicating the correlation between each intervertebral disc and the predicted Pfirrmann classification

*IVD* intervertebral disc, *PF* Pfirmann

## Discussion

To the best of our knowledge, this study is the first to evaluate the use of a complex CNN–GNN data pipeline for the analysis of degenerative disc disease on lumbar MRI images.

Our results for inter-rater reliability according to the Pfirrmann grading system exhibited moderate inter-rater agreement, with Cohen’s kappa values in the range 0.455–0.565. This finding is partially consistent with those of previous studies. For instance, Niemeyer et al. demonstrated a similar level of inter-rater reliability in the human Pfirrmann classification of discs, with a kappa value of 0.59, aligning with our moderate agreement category [7]. However, Carrino et al. exhibited a slightly higher kappa value of 0.66, which fell within the substantial agreement range [3].

The lowest inter-rater reliability was observed in the L1/2 segment, whereas the highest reliability was observed in the L5/S1 segment. Regarding the L1/2 segment, the GNN showed a low performance at the segmental level. However, its performance in the L5/S1 segment was unsatisfactory. Teraguchi et al. conducted a cross-sectional study on the prevalence and distribution of disc degeneration and showed that in the lumbar spine, the L1/2 (30.85%) segment exhibited the least degenerative changes, whereas the L4/5 (72.45%) and L5/S1 (68.8%) segments exhibited the most severe signs of degeneration [24]. The poorer inter-rater reliability may be attributed to the minor changes observed on MRI with mild degenerative changes in the L1/2 segment that are difficult to differentiate by the human eye.

Our study's results can be further contextualized within the broader landscape of automated IVD analysis. Jamaludin et al. achieved high accuracy in disc localization and segmentation using a vertebral approach [25]. Castro-Mateos et al. focused on 2D segmentation and degeneration grading [26], achieving promising results but lacking the 3D perspective our method provides. Huang et al.'s Spine Explorer demonstrated the potential of deep learning for vertebrae and disc quantification, aligning with our goal of comprehensive spine analysis [27].

It's important to note that the Pfirrmann system poses certain challenges for deep learning models, as observed by Gao et al. [28]. Their push–pull regularization network addressed some of these challenges, achieving high accuracy in automated grading. Our GNN-based approach aims to tackle these difficulties by incorporating 3D structural information, potentially offering a more comprehensive analysis of disc degeneration.

A recent study by Soydan et al. used a similar U-Net segmentation approach followed by Inception V3 classification, achieving high accuracy (segmentation: 0.9597, classification: 0.9346) [29]. While our segmentation performance was slightly lower (F1 score: 0.85), both studies demonstrate the potential of CNN-based approaches in IVDD analysis.

GNNs sometimes struggle with overfitting the training data, which can reduce their performance on new, unseen data. To mitigate this, we implemented several strategies, including data augmentation techniques, a weighted loss function to address class imbalance, and decoupled weight decay regularization during training. These steps aim to improve the model's generalization capability and robustness to unseen data.

This study had several limitations. The training data for this study, despite encompassing a wide range of patient ages, had most patients averaging approximately 60 years. This age distribution could potentially lead to underrepresentation of lower Pfirrmann grades in the dataset. Moreover, as this was a single-center study, the findings were confined to different MRI scanners, substantially limiting the generalizability of the results.

Our current dataset and study design did not allow for analysis of potential gender or BMI effects on model performance. Previous research has shown that these factors can influence disc degeneration patterns [30]. Future studies should consider stratifying results by gender and BMI, and potentially incorporating these as input features to improve model accuracy and generalizability.

The potential for employing 3D models in IVD assessment is a promising avenue for future research but requires validation. Our findings support the integration of AI tools with human expertise for more accurate evaluation of spinal pathology. However, the limitations of this study, such as potential biases in image selection and inconsistencies in the GNN model, must be acknowledged. These factors may affect the generalizability of our results, underscoring the importance of further research using larger and more diverse datasets to establish the effectiveness of new classification systems, particularly those that utilize 3D evaluation techniques.

## Conclusion

Our study introduces a novel CNN-GNN pipeline for 3D visualization and classification of lumbar disc degeneration. While the model’s performance varied across spinal levels, it demonstrated the potential of integrating 3D geometric information in automated IVD analysis. Despite current limitations, this approach offers a new perspective on capturing spatial relationships in disc degeneration assessment. Future research should focus on refining these techniques, utilizing larger datasets, and incorporating additional clinical factors to improve accuracy and generalizability. The continued development of such AI-based 3D imaging approaches could significantly enhance our understanding and evaluation of intervertebral disc degeneration.

## Data Availability

The detailed datasets used in this study are available from the corresponding author upon request.

## Abbreviations

MRI: Magnetic resonance imaging
CNN: Convolutional neural network
GNN: Graph neural network
ML: Machine learning
IVD: Intervertebral disc
VB: Vertebral body
AI: Artificial intelligence
3D: Three-dimensional
LBP: Low back pain
